# Editorial: Co-Infection and Consequences in Cystic Fibrosis

**DOI:** 10.3389/fcimb.2022.924527

**Published:** 2022-06-01

**Authors:** Barbara C. Kahl, Karen Moreau

**Affiliations:** ^1^ Institute of Medical Microbiology, University Hospital Münster, Münster, Germany; ^2^ CIRI, Centre International de Recherche en Infectiologie, Université de Lyon, Inserm, U1111, Université Claude Bernard Lyon 1, CNRS, UMR5308, Lyon, France

**Keywords:** cystic fibrosis, microbiome, co-infection, bacterial interaction, evolution and adaptation, host response, clinical outcome

Cystic fibrosis (CF) is the most common inherited disease in the Caucasian population with app. 70,000 individuals affected worldwide (Elborn, 2016). This disease is linked to a mutation in the *cftr* gene, resulting in alteration of the CFTR protein, a chloride channel located at the apical site of epithelial cells in many organs (Zielenski et al., 1991). At the pulmonary level, the mutation is responsible for the presence of viscous mucus which inhibits ciliary beat. The modification of the pulmonary environment and the alteration of mucociliary clearance promote the implantation and multiplication of specific pathogens linked to CF and thus cause a modification of the pulmonary microbiota (Hauser et al., 2011; Egan, 2016; Saint-Criq and Gray, 2017; French Cystic Fibrosis Register, 2017; Foundation ACF, 2020; Society ECF, 2020). Chronic bacterial respiratory infections in people with CF (pwCF) are responsible for progressive lung disease with exacerbations and bronchiectasis leading to morbidity and mortality with a reduced life expectancy of 46 years. It is estimated that 80% of deaths are due to bacterial infections of the lungs (Turcios, 2020).

In the CF modified lung environment, mostly specific microbial species (*Pseudomonas aeruginosa, Staphylococcus aureus, Stenotrophomonas maltophilia, Burkholderia cepacia complex, Achromobacter xylosoxidans, Exophiala dermatitidis, Aspergillus fumigatus* and *some viruses*) are found and presumably can interact synergistically or antagonistically (Boutin et al., 2015; Bisht et al., 2020; Khanolkar et al., 2020; Eichelberger and Cassat, 2021). These interactions themselves generate changes in the composition of the pulmonary microbiota and may also modify the adaptation and virulence of certain pathogens.

The clinical consequences of polymicrobial infections are of major interest. Understanding the mechanisms of microbial interactions and their clinical relevance is essential to rationally orient current therapies and to develop improved treatments for respiratory infections. Deciphering the impact of co-infection and microbial interactions on the host response at the cellular and molecular levels remains a largely unexplored field. The same is true for the role and impact of polymicrobial infections on the evolution and adaptation of pathogens in the context of chronic infection/colonization.

This Research Topic begins with a very nice review by Biswas and Götz in which they discuss the complexity of bacterial interactions in the context of polymicrobial infection. They describe the competitive and cooperative interactions of the two major CF pathogens, *S. aureus* and *P. aeruginosa*, and the molecular mechanisms involved. We are thus discovering that by focusing on only two bacterial species, multiple interactions can be set up.

However, the pulmonary microbiome of cystic fibrosis patients is much more complex and evolves with the age of the patients. Metzger et al. address the question of the link between diversity and dynamic of the microbiome and the decline in respiratory function. By following the composition of the lung microbiome of 12 adolescents for 3 to 5 years, they show that the presence of a diverse and dynamic microbiome is associated with a less severe decline in lung function. Conversely, the presence of a stable, low diversity microbiome dominated by Bacteroidetes and Firmicutes appears to be a major contributor to the severity of the infection. The protective role of a diverse microbiome is reinforced by the study of Tony-Odigie et al. By exploring *in vitro*, the impact of commensal bacterial species on the inflammatory response induced by *P. aeruginosa*, they show that several commensal bacteria exhibit a protective effect, including certain strains of *Streptococcus mitis*. The simultaneous presence of *S. mitis* and *P. aeruginosa* reduces the activation of pro-inflammatory signaling pathways in pulmonary epithelial cells. Comparative genomic analysis of *S. mitis* strains has identified potential genes involved in this protective role. These recent findings pave the way for numerous studies aimed at understanding how the diversity of the lung microbiome influences the host response.

In order to explore this question Graf et al. propose a novel protocol for metaproteomic analysis of CF patient sputum. This protocol allows to analyze the physiology of bacteria as closely as possible to *in vivo* conditions and may allow to identify the metabolic pathways involved and essential in a polymicrobial context.

In summary, the articles in this Research Topic discuss important features of the complexity of the lung microbiome in cystic fibrosis patients, its evolutionary dynamics and its impact on the evolution of lung function. The field of research concerning these issues is vast and promises many more major scientific studies.

## Author Contributions

All authors listed have made a substantial, direct, and intellectual contribution to the work and approved it for publication.

## Conflict of Interest

The authors declare that the research was conducted in the absence of any commercial or financial relationships that could be construed as a potential conflict of interest.

## Publisher’s Note

All claims expressed in this article are solely those of the authors and do not necessarily represent those of their affiliated organizations, or those of the publisher, the editors and the reviewers. Any product that may be evaluated in this article, or claim that may be made by its manufacturer, is not guaranteed or endorsed by the publisher.
